# Mitochondrial Aging in the CNS: Unravelling Implications for Neurological Health and Disease

**DOI:** 10.3390/biom15091252

**Published:** 2025-08-29

**Authors:** Davide Steffan, Camilla Pezzini, Martina Esposito, Anais Franco-Romero

**Affiliations:** 1 Department of Biomedical Sciences, University of Padova, Via Ugo Bassi 58/B, 35131 Padova, Italy; davide.steffan@unipd.it (D.S.); camilla.pezzini@unipd.it (C.P.); martina.esposito.2@phd.unipd.it (M.E.); 2 Veneto Institute of Molecular Medicine, Via Orus 2, 35129 Padova, Italy

**Keywords:** aging, mitophagy, CNS, neurodegenerative diseases

## Abstract

Mitochondrial aging plays a central role in the functional decline of the central nervous system (CNS), with profound consequences for neurological health. As the brain is one of the most energy-demanding organs, neurons are particularly susceptible to mitochondrial dysfunction that arises with aging. Key features of mitochondrial aging include impaired mitochondrial dynamics, reduced mitophagy, increased production of reactive oxygen species (ROS), and accumulation of mitochondrial DNA (mtDNA) mutations. These alterations dramatically compromise neuronal bioenergetics, disrupt synaptic integrity, and promote oxidative stress and neuroinflammation, paving the path for the development of neurodegenerative diseases. This review also examines the complex mechanisms driving mitochondrial aging in the central nervous system (CNS), including the disruption of mitochondrial-organelle communication, and explores how mitochondrial dysfunction contributes to neurodegenerative diseases, such as Alzheimer’s, Parkinson’s, Huntington’s, and amyotrophic lateral sclerosis. By synthesizing current evidence and identifying key knowledge gaps, we emphasize the urgent need for targeted strategies to restore mitochondrial function, maintain cognitive health, and delay or prevent age-related neurodegeneration.

## 1. Introduction

Aging involves a gradual decline of an organism’s functional status, leading to an increasing susceptibility to develop pathologies, as well as a higher risk of death with chronological age. This deteriorating process affects all tissues, including the central nervous system (CNS) and the brain, which is particularly vulnerable to the cumulative effects of cellular stress and metabolic dysfunction. Understanding the cellular and molecular mechanisms that drive age-associated decline has become a central focus of biomedical research.

Among these mechanisms, mitochondrial dysfunction has emerged as a key contributor to aging and neurodegeneration. Mitochondria, often referred to as the “powerhouses” of the cell, are essential for ATP production through oxidative phosphorylation, regulation of calcium homeostasis, and control of apoptotic pathways. However, during aging, mitochondrial function progressively deteriorates. This decline is characterized by increased production of reactive oxygen species (ROS), accumulation of mitochondrial DNA (mtDNA) mutations, impaired dynamics (fission and fusion), and reduced mitophagy, the selective removal of damaged mitochondria.

In the CNS, where neurons are highly energy-dependent and possess limited regenerative capacity, mitochondrial health is critical for maintaining synaptic function, neurotransmission, and overall cellular homeostasis. As a result, age-related mitochondrial dysfunction exacerbates neuronal vulnerability, and it is strongly implicated in the pathogenesis of several age-related neurodegenerative diseases, including Alzheimer’s disease, Parkinson’s disease, and amyotrophic lateral sclerosis.

Emerging evidence also highlights a complex interplay between mitochondrial signaling and inflammation in the aging brain. Damaged mitochondria can release mtDNA and other danger-associated molecular patterns (DAMPs) into the cytosol or extracellular space, triggering innate immune responses and contributing to chronic neuroinflammation another hallmark of neurodegenerative conditions.

Thus, mitochondria are not only central to cellular metabolism but also play a pivotal role in determining the fate and function of neural cells during aging. Our goal is to provide a comprehensive and up-to-date overview of mitochondrial defects during ageing that might trigger neurodegenerative diseases pathogenesis. For this we conducted a literature search using the PubMed database, focusing on recent publications from 2020 to 2025, supplemented by key earlier studies. Search terms included “mitochondria and aging” “CNS and aging,” “mitophagy,” “neurodegeneration,” and specific disease-related terms (such as “ALS and mitochondria”). Searches were refined for each topic to explore mechanisms in depth. We prioritized peer-reviewed studies based on scientific rigor and relevance for this review. This review will elucidate not only the different mechanisms linking mitochondrial dysfunction and CNS diseases but also highlight mitochondrial molecular targets that could potentially be modulated as therapeutic intervention to preserve cognition and delay neurodegeneration.

## 2. Mitochondrial Quality Control During Aging in CNS

The maintenance of a healthy mitochondria pool through finely tuned mitochondria turn-over processes, namely, mitochondria degradation and biogenesis, is crucial to maintain optimal mitochondrial function and, consequently, neuronal health and viability. Indeed, in physiological conditions, mitochondria are constantly kept under strict quality-control. However, a decrease in a cell’s catabolic capacity and thus ineffective removal of non-functional and damaged subcellular components, contributes to the onset and progression of brain aging and the development of several pathological conditions. Consistently, mitochondrial quality progressively declines with aging and is extremely exacerbated in age-related neurological diseases [1,2].

### 2.1. Decline in Mitophagy During Aging

In mammals, the clearance of damaged mitochondria, both under physiological conditions and in response to cellular stressors, is primarily achieved through selective autophagy, known as mitophagy. Mitophagy, first identified in 2005 by Lemasters et al. [3], is a core cellular quality-control process that safeguards the functionality of the mitochondrial network and allows its adaptation to different stimuli [4,5].

Mitophagy can be elicited through two different pathways: the PINK1/Parkin-dependent pathway, and the receptor-mediated, PINK1/Parkin-independent mechanism [6]. The best-characterized of these is the ubiquitin-mediated pathway, orchestrated by the mitochondrial kinase PINK1 (PTEN-induced putative kinase 1) and the cytosolic E3 ubiquitin ligase Parkin (PARK2). Upon mitochondrial damage and loss of membrane potential, PINK1 accumulates on the outer mitochondrial membrane (OMM) and becomes activated, triggering a phosphorylation cascade. A key step involves the PINK1-mediated phosphorylation of Parkin [6], which, in turn, activates Parkin and promotes its translocation to the OMM. Once there, Parkin ubiquitinates multiple outer membrane proteins, such as mitofusin-1/2 (Mfn1/2) and dynamin-related protein 1 (Drp1) [7,8], targeting the mitochondria for degradation. Ubiquitinated mitochondria are subsequently recognized by autophagy adaptor proteins, including p62/SQSTM1, optineurin (OPTN), NDP52, NBR1, and TAX1BP1 [9,10], which facilitate mitochondrial engulfment by autophagosomes.

The involvement of PINK1/Parkin signaling is particularly noted in the context of neurodegenerative diseases. Their levels are downregulated in Alzheimer’s, Parkinson’s, and amyotrophic lateral sclerosis (ALS), and are accompanied by aberrant mitophagy and/or impaired mitophagy flux in human brains [11,12,13]. However, in mice tissues or cells of high metabolic demand, the loss of PINK1 is not sufficient to alter basal mitophagy including microglia [14], suggesting that other pathways may contribute to basal mitophagy process in these contexts. Moreover, neither PINK1 or Parkin knockout mice exhibit clear neurodegenerative phenotypes under normal physiological conditions [15].

Notably, despite their cooperation in stress responses, PINK1 and Parkin have distinct subcellular localizations, and may perform independent functions under basal conditions [16]. For example, PINK1 is believed to phosphorylate a broader set of substrates beyond Parkin, while Parkin activation can also be influenced by aging-related factors and PINK1-independent signaling pathways [17].

Beyond the ubiquitin-dependent mechanism, PINK1/Parkin-independent mitophagy plays a crucial role in maintaining mitochondrial health under normal conditions. Studies in PINK1-deficient mice [14] and *Drosophila* lacking either PINK1 or Parkin [18] have shown that basal mitophagy can occur independently of mitochondrial depolarization and ubiquitin signaling, underscoring the existence of alternative receptor-mediated pathways [18,19,20]. These pathways are mediated by a range of mitophagy receptors that localize predominantly to the OMM and contain an LC3-interacting region (LIR) motif, enabling direct interaction with autophagosomal proteins LC3 and GABARAP. Key examples include FUN14 domain-containing 1 (FUNDC1), Autophagy and Beclin 1 Regulator 1 (AMBRA1), B-cell lymphoma 2 (BCL2)/adenovirus E1B 19-kDa-interacting protein 3-like (BNIP3L/NIX), and its homolog BCL2/adenovirus E1B 19-kDa-interacting protein 3 (BNIP3) [6]. Interestingly, Schmid and colleagues recently found that activation of BNIP3 in *Drosophila* brain tissue could reverse the accumulation of dysfunctional mitochondria across aging by increasing mitophagy, resulting in improved organism lifespan [21]. However, while BNIP3 activation in *Drosophila* shows promising effects on mitophagy and lifespan, caution is needed when extrapolating these findings to humans, given the significant physiological differences between invertebrates and mammals. Further studies in vertebrate models are needed to assess the translational potential.

**Figure 1:**
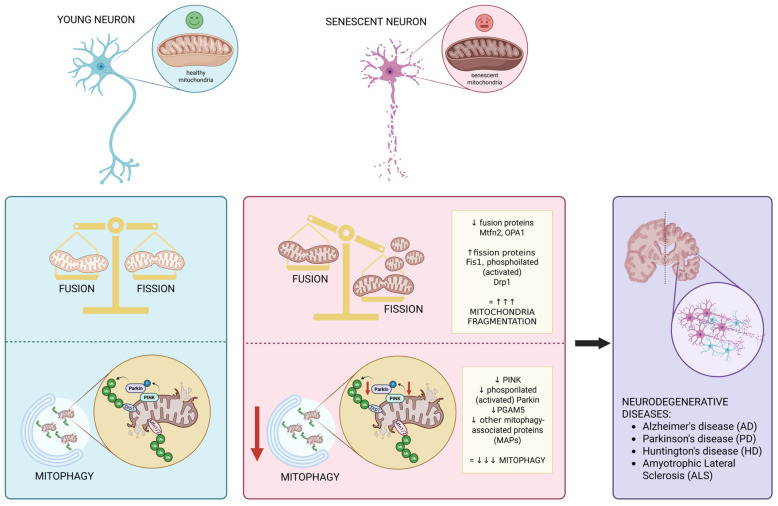
Alterations in mitochondrial dynamics and mitophagy impairment in neuronal senescence and neurodegeneration. Scheme representing alterations in mitochondrial dynamics and mitophagy in senescent neurons compared with younger neurons. In young neurons mitophagy and mitochondrial dynamics are balanced. In senescence cells, decreased fusion proteins (OPA1, Mtfn2) and increased fission proteins (Drp1 and Fis1) triggers mitochondria fragmentation. Moreover, mitophagy is also impaired due to decreased PINK1 and PGAM5 expression, lower activation of Parkin and less mitophagy-associated proteins (MAPs) expression. Those alterations during aging might trigger neurodegenerative diseases, such as Alzheimer’s, Parkinson’s, Huntington’s, and amyotrophic lateral sclerosis. Image created with Biorender (https://www.biorender.com/, accessed on 27 July 2025).

As previously mentioned, an established consequence of altered mitophagy is cellular senescence. Cellular senescence is a critical feature of aging and has been recognized as the primary component of age-associated neurodegenerative disorders [22]. Indeed, excessive senescence can have detrimental effects and cause or contribute to different aging-associated phenotypes and diseases [23]. It is noteworthy that prevalent neurodegenerative diseases in humans, such as Alzheimer’s, Parkinson’s, and ALS, are accompanied by aberrant mitophagy and/or impaired mitophagy flux [12] and this is paralleled by a reduced presence of specific markers, such as PINK1, Parkin, phosphoglycerate mutase 5 (PGAM5), and other already-mentioned mitophagy-associated proteins (MAPs), in the plasma from patients with several types of dementia, cognitive impairment or neurodegenerative diseases, reflecting a significant downregulation of this pathway in these pathological conditions [13,17] (Figure 1). Moreover, the maintenance of mitophagy in order to blunt the dramatic decline observed with aging can be exploited pharmacologically to modulate this biological process with the aim to counteract the progression of neurodegeneration and preserve a healthy lifespan. Indeed, supplementation with chemical inducers of mitophagy has been shown to mitigate cognitive deficits in different models of Alzheimer’s disease [24,25]. Examples of pharmacological agents and phytochemicals that have been proposed as mitophagy promoters acting on different molecular targets include resveratrol, curcumin, spermidine, taurine, catechins, and melatonin [26,27,28,29]. On the other hand, there are also reported cases where uncontrolled mitophagy proves detrimental and aggravates neuronal cell death [30,31,32,33].

The complexity of mitophagy regulation is further underscored by the prominent differences that have been characterized in basal mitophagy activity in neurons of different brain regions in vivo [34]. Moreover, although less extensively studied, mitophagy in glial cells is an emerging area of interest, with growing recognition of its potential physiological and pathological relevance [7]. For example, dysfunction in mitochondrial metabolism in astrocytes, which are responsible for nearly 20% of the brain’s total oxygen consumption through oxidative phosphorylation within their mitochondria [35], can lead to decreased neuronal activity and contribute to the development of neurodegenerative diseases [36]. Moreover, most astrocytes become senescent during aging [37,38]; therefore, it is likely that astrocytic senescence participates in the initiation and progression of neurodegenerative disease, including Parkinson’s disease [39,40]. Importantly, senescent astrocytes exhibit increased mitochondrial fragmentation and impaired mitophagy coupled with upregulated mitochondrial biogenesis, leading to the accumulation of damaged mitochondria that are not efficiently cleared [41]. Recently, Chen et al. [42] have proposed a small aminopropyl carbazole compound, P7C3, as a new neuroprotective compound, capable of suppressing the increased production and secretion of senescence-associated secretory phenotype (SASP) factors exhibited by senescent astrocytes in vitro. This occurs through concomitant promotion of mitophagy and inhibition of mitochondrial ROS generation. This is particularly relevant since the massive release of chemokines and proinflammatory cytokines from senescent astrocytes can lead to the death of surrounding neurons through inflammatory damage, loss of neuroprotective functions, impaired synaptic plasticity, and induced glutamate cytotoxicity [42,43].

Collectively, these studies strongly suggest that mitophagy must be tightly regulated to preserve neuronal function along with aging, since its dysregulation, whether increased or decreased, can affect brain homeostasis promoting neuropathology and neurodegeneration.

### 2.2. Dysregulation of Mitochondrial Dynamics During Aging in CNS

Mitochondria form an interconnected network within the cell, and homeostasis of this network greatly affects cellular function and survival. Moreover, finely tuned mitochondrial dynamics allow effective adaptation of these organelles in response to various cellular needs and environmental cues [44,45]. Indeed, it can be assumed that maintaining a balance between the two opposing processes, mitochondrial fission and fusion, is an emerging essential factor of longevity [46]. Mitochondrial fission is essential for mediating stress response, metabolic regulation, and mitophagy, but excessive levels can result in fragmented and defective mitochondria. On the other hand, fusion allows a constant mixing and exchange of proteins, metabolites, and mtDNA between mitochondria, which can be beneficial, leading to restoration of mitochondrial function [1,47]. Indeed, mitochondrial fusion promotes oxygen consumption, oxidative phosphorylation, and, consequently, increased ATP production [48,49]. As organisms age, mitochondrial fission and fusion become dysregulated and mitochondrial networks become fragmented [44,50,51,52]. Furthermore, dysregulated expression of fission and fusion proteins occurs in both aging and age-related diseases [53,54,55] (Figure 1). Coherently, modulation of mitochondrial dynamics has been shown to affect longevity in different species. Notably, increased mitochondrial fusion has been associated with increased longevity [56]. Healthy human centenarians have highly connected mitochondrial networks compared to younger individuals [57]. Moreover, it has long been demonstrated that mitochondrial fusion impairment resulting from decreased mitofusin expression is associated with impaired cell growth and respiration, an heterogeneous mitochondrial population, and decreased membrane potential [58]. These effects are characteristic of brain aging and, in particular, may play a detrimental role by damaging neighbouring neuronal precursors [46]. Indeed, some studies propose a link between aging and stem cell loss in the CNS [59,60], although the causes of this phenomenon have not yet been elucidated.

On the other hand, activated mitochondrial fission can be beneficial for the elimination of defective mitochondria fragments accumulated during aging. However, it has been suggested that increased expression of mitochondrial fission proteins may disrupt the mitochondrial membrane potential and inhibit the respiratory chain, leading to slower cell growth and exacerbated neuronal aging [46]. On the contrary, mitochondrial fission inhibition is sufficient to increase lifespan in yeast and fungal models [61,62]. In particular, deletion of dynamin-related protein 1 (Drp-1) increases lifespan and restores motility in a neuronal model of polyglutamine toxicity [63]. Moreover, studies in *C. elegans* have shown that promoting mitochondrial fusion by deleting Drp-1 amplifies the longevity-promoting effects of dietary restriction model *daf-2*, but not in wild-type [64,65]. Moreover, it has been observed that the amount of the activated (phosphorylated) form of Drp1 increases with aging [66]. In particular, phosphorylation of Drp1 at Serine 616 (S616) activates Drp1 and results in mitochondrial fragmentation, and is possibly involved in neuronal death [67], suggesting that the increased level of phospho-Drp1 correlates with the mitochondrial morphological changes in the hippocampus and the cognitive decline observed with aging [46]. Consistently, Li Y. et al. demonstrated that murine hippocampus and cerebral cortex aging processes are accompanied by significant increase in fission proteins Fis1 and Drp1 [68], which also contributes to the mitochondrial fragmentation activation (Figure 1).

Importantly, dysregulated mitochondrial fission was observed in inflamed microglia [69,70], whereas mitochondrial division inhibitor 1 (mdivi-1) and Drp1 knockdown inhibited mitochondrial fission, mitochondrial ROS generation, and proinflammatory mediators in LPS-stimulated microglia [71]. Indeed, mitochondrial homeostasis disruption contributes to microglia-mediated inflammation and vice versa. Moreover, the natural compound atractylenolide III was reported to suppress neuroinflammation and protect against brain ischemia by inhibiting JAK2/STAT3-dependent mitochondrial fission in microglia [72].

Finally, it has recently been shown that the simultaneous overexpression of proteins regulating both mitochondrial fission and fusion despite (or perhaps due to) an increased level of mitochondrial fragmentation in *C. elegans* was positively correlated with stress tolerance and longevity [73].

All these evidences, together emphasize the relevance of developing therapeutic approaches that would maintain mitochondrial dynamics balance in order to actively improve health and lifespan, as well as to counteract CNS aging and neurodegenerative diseases development.

## 3. Energy Demands of the CNS During Aging

The brain is one of the most highly energy-demanding organs, consuming almost 20% of the organism’s total oxygen and glucose [74]. Brain functions depend heavily on mitochondria activity, as they generate adenosine triphosphate (ATP), which serves as the fuel of several neuronal cell types to exchange signals with other neurons through synapses [75]. Neuronal energy production primarily relies on mitochondrial processes (Krebs cycle and oxidative phosphorylation). Pyruvate from glycolysis enters mitochondria, forming acetyl-CoA for the Krebs cycle. NADH from the Krebs cycle fuels ATP production via the oxidative phosphorylation system (OXPHOS), which involves electron transfer between the respiratory chain complexes (from I to IV), together with the transport of protons across the mitochondrial membrane. This process leads to the synthesis of ATP by the ATP synthase complex. In addition to this, neurons can generate a small amount of ATP from aerobic and anaerobic glycolysis in the cytoplasm.

OXPHOS is also the main energy source for both microglia and oligodendrocytes under normal conditions. However, under specific neurological or pathological conditions, both cell types can shift toward aerobic or anaerobic glycolysis to meet their energy needs [76,77,78].

On the other hand, astrocytes display a highly glycolytic metabolism, which results in the conversion of glucose into lactate, with low oxygen consumption; lactate is then transported to neurons where it is fully oxidized to produce ATP [79,80]. In addition, astrocytes have a high mitochondrial content, which helps buffer intracellular calcium levels important for modulating neurotransmitter signaling in these cells. Mitochondria in astrocytes also support various energy-demanding processes involved in neuron-glia communication.

Overall, glucose is the main source of energy in the brain, while ketone body metabolism is an optional source of energy in case of lack of glucose [81].

Mitochondria located in neurons axons provide the ATP necessary for impulse conduction; therefore, their trafficking must be tightly regulated in synaptic terminals to meet the demands of multiple synaptic activities [82]. The axonal excitability sustaining the impulse conduction relies on ion channels and energy-dependent pumps that generate action potentials along the axon. Sodium and potassium pumps are essential to maintain membrane resting potentials by coupling ATP hydrolysis together with ionic translocation across the mitochondrial membrane.

Physiological cell activity is the result of a combination of established and maintained bioenergetic homeostasis. In the brain, this equilibrium is perturbed during aging, when there is an evident decline in energy metabolism [83].

The ability of aging neurons to sustain axon sprouting and growth can be influenced by any alteration in OXPHOS and the electron transport chain (ETC) [84]. A decline in the activity of all mitochondrial ETC complexes has been found in aged brains of rodents and primates [85,86].

A single-cell transcriptome atlas of the aged brain of *Drosophila melanogaster* reported a sustained decline of OXPHOS-related genes across its lifespan [87]. Furthermore, it has been reported that in aged rat brain a significant reduction in electron transfer is also associated with a decreased mitochondrial inner membrane potential [52]. Moreover, a significant decline in the NAD+/NADH ratio, products of OXPHOS and glycolysis, pyruvate decarboxylation, and the Krebs Cycle, supports the reduction in ETC enzyme activity in mitochondria with age [88], thus making it a potential therapeutic target for several age-dependent pathologies [89]. Taken together, alterations of the mitochondrial machinery may underlie overall hypometabolism and increased oxidative stress. Glucose metabolism alterations promote reduced catabolic activity, which is commonly linked to aging in many brain regions. Because of the brain’s constant demand for energy, age-associated metabolic impairments may have significant consequences for normal brain function, particularly for neurons, ultimately resulting in cognitive deficits.

A recent study showed a molecular model of the neuro-glia-vascular system, capturing the complex interactions between brain metabolism, blood flow, and neuronal activity during aging using RNA sequencing data [90]. This model reveals a reduced metabolic flexibility in the aging brain, highlights astrocytes’ role in supporting neuronal stability, and identifies potential anti-aging targets, including enhancement of the NADH cytosol–mitochondria shuttle, NAD+ pool, lactate, and Na+/K+ ATPase, while also proposing the reduction in blood glucose levels. The study provides an open-source tool to advance research on neurodegenerative diseases.

Another recent study highlights that levels of succinylation, a novel post-translational modification (PTM) involved in regulating mitochondrial energy metabolism pathways, increase with aging [91]. Particularly, succinylation enhances glycolysis, increases mitROS production, and triggers neuroinflammation. Downregulation of this PTM through administration of succinyl phosphonate (SP) showed improvement of neuroinflammation in vivo suggesting that targeting this pathway may be important for treating aging-related neuroinflammation [91].

In conclusion, therapeutic strategies aimed at rebalancing OXPHOS and glycolysis, along with antioxidant treatment, may help delay neurodegeneration [92].

## 4. Mitochondrial ROS Production and Oxidative Stress in the CNS

Mitochondria are a major source of oxidative stress in neurons. Indeed, OXPHOS is not only essential for ATP production, but it also generates ROS such as superoxide anions and hydrogen peroxide, particularly at Complexes I, II, and III [93]. These ROS contribute to redox imbalance, neurotoxicity, genomic instability, and inflammation [94]. Due to their unpaired electrons, ROS possess strong oxidative potential, damaging nuclear and mitochondrial DNA, lipids, and proteins. This cumulative damage impairs mitochondrial function and progressively affects postmitotic neurons over time, a process that worsens with aging and is closely associated with neurodegenerative diseases, such as Alzheimer’s and Parkinson’s, as well as cancer [95].

The aging theory suggests that mtDNA damage accumulates over time, depleting NAD+ and increasing oxygen consumption and ATP production. This leads to mitochondrial hypercoupling, elevated membrane potential, increased production of free radicals, and decreased mitophagy. The cycle continues as free radicals cause further DNA damage [96]. The impact of ROS in mitophagy has been studied and might be dose-dependent. At lower levels, ROS activate mitophagy by stabilizing PINK1 and recruiting PARKIN, which is beneficial for removing defective mitochondria. Conversely, at higher levels, ROS can S-nitrosylate PARKIN, inhibiting its function and leading to mitochondrial dysfunction, protein misfolding, synaptic impairment, and cell death [97,98].

A recent study also demonstrated that ROS are involved in dysregulating the SMO-SHH-GLI signaling pathway [99]. The Sonic Hedgehog (SHH) pathway is important for cell division, cellular differentiation and the maintenance of neuronal integrity. It has shown neuroprotective effects in many age-related diseases, such as Parkinson’s and Alzheimer’s disease. Dysregulation of the SMO-SHH-GLI axis is linked to the proteolytic cleavage of GLI (glioma-associated oncogene homolog) into GLI3 (a repressor), which suppresses the expression of neurotrophic factors such as brain-derived neurotrophic factor (BDNF), increasing vulnerability to oxidative stress and triggering neuronal damage [100].

To counteract the ROS-induced oxidative damage, cells activate several antioxidant defences, including superoxide dismutase (SOD), catalase (CAT), and glutathione peroxidase (GSx), to maintain redox balance [101]. Given the brain’s critical function, its antioxidant system must operate efficiently to prevent oxidative stress, making the brain particularly vulnerable to ROS-related damage. In the aging human brain, particularly in the hippocampus and frontal cortex, there is a progressive increase in protein nitration and oxidation, accompanied by a decline in the activity of antioxidants enzymes such as SOD and glutathione (GSH) [102]. For these reasons, the use of antioxidants such as glutathione has emerged as a promising therapeutic approach to mitigate neuroinflammation and prevent neuronal damage [103].

In parallel, recent research suggests that caloric restriction might enhance the resilience of the central nervous system to neurodegenerative pathologies, most of which occur during aging, possibly by reducing ROS production and boosting neurotrophic factors and chaperones [104,105,106]. These studies underscore the potential of antioxidant-based therapies as effective tools in addressing age-related brain disorders.

## 5. Mitochondrial DNA Mutations During Aging

Unlike nuclear DNA (nDNA), mitochondrial DNA (mtDNA) is polyploid, with each mitochondrion containing multiple copies of mtDNA. Consequently, mutations may be present in some, but not all, copies, a phenomenon known as heteroplasmy. mtDNA replication occurs both in coordination with cell division (strict replication) and independently of the cell cycle (relaxed replication). Furthermore, mtDNA is organized into nucleoprotein complexes called nucleoids, which are typically positioned near the inner mitochondrial membrane [107].

mtDNA is significantly more susceptible to damage and mutation than nDNA [108]. This increased vulnerability is attributed to several factors: its proximity to ROS generated during oxidative phosphorylation, the absence of protective histones, a high replication frequency, and a limited amount of non-coding DNA, which increases the likelihood that mutations will affect essential genes [109].

The accumulation of mtDNA damage has been implicated in a wide spectrum of pathological conditions, including neurodegenerative diseases, cancer, and metabolic disorders [110]. These mutations can be maternally inherited or acquired somatically. Typically, point mutations are more prevalent in mitotically active tissues, whereas large-scale deletions preferentially accumulate with age in post-mitotic cells such as neurons and muscle fibers [111,112,113], where they can reach levels that impair respiratory chain function. For instance, deletion loads exceeding 50% have been reported in dopaminergic neurons of the substantia nigra, which are closely associated with the pathogenesis of neurodegenerative diseases [114,115].

Contrary to earlier beliefs that damaged mtDNA is simply degraded, current evidence supports the presence of active DNA repair mechanisms within mitochondria. Among these, base excision repair (BER) is the most extensively characterized and is responsible for correcting small base lesions, such as oxidized or deaminated nucleotides. This pathway is initiated by DNA glycosylases, including both mono-functional (e.g., AAG, MUTYH, UNG) and bi-functional enzymes (e.g., OGG1, NEIL1/2, NTHL1), which excise damaged bases to generate abasic sites. These are then processed by APE1 and PNKP, while DNA polymerase γ and ligase III complete the repair process [116,117]. Other proposed repair pathways include direct reversal (DR) by enzymes such as MGMT, mismatch repair (MMR), potentially involving YB-1, and double-strand break repair (DSBR) via homologous recombination (HR) or non-homologous end joining (NHEJ), although their functional relevance in mitochondria remains less defined.

Moreover, mitochondrial fusion and fission help manage mtDNA damage: fusion dilutes harmful mutations by mixing contents, while fission isolates damaged components for removal via mitophagy. This clearance supports mitochondrial quality control and influences cellular signaling, including activation of the cGAS-STING pathway, thereby linking mtDNA damage to inflammation and innate immunity [118,119].

Nonetheless, the mechanisms by which individual mtDNA deletions clonally expand to dominate within a cell remain poorly understood. Although stochastic models, such as that proposed by Elson et al. [120] predict only ~4% of cells would become respiratory-deficient by age 80, empirical data suggest much higher rates: up to 40% of neurons deficient in cytochrome c oxidase (COX) activity [121,122]. Several hypotheses have been proposed to explain this discrepancy. One suggests that deleted genomes may have a replicative advantage due to their smaller size [123,124]. Another model proposes a compensatory replication response, in which the loss of mtDNA-encoded proteins triggers replication to restore mitochondrial function [125]. However, this explanation is challenged by the heterogeneous nature of deletion breakpoints.

A more recent and intriguing hypothesis is the “perinuclear niche” model in skeletal muscle [126], which proposes that deletions occurring in mitochondria near the nucleus may trigger retrograde signaling events, such as changes in ATP/ADP or NAD+/NADH ratios, that stimulate local mtDNA replication. This localized replication could account for the patchy distribution of mitochondrial dysfunction observed in skeletal muscle. Whether this mechanism operates similarly in neurons remains uncertain, given their distinct mitochondrial architecture and dynamics [127,128,129].

As with point mutations, inherited mtDNA deletions are often purged from mitotically active tissues such as blood [130], but they persist, or even expand, in long-lived post-mitotic tissues, including muscle and brain [131,132]. These observations underscore the critical role of tissue context in shaping mtDNA deletion dynamics and their pathological consequences.

## 6. Mitochondrial-Nuclear Communication and How This Affects Health and Neuronal Function During Aging

### 6.1. Mitochondria–Nuclear Communication

Mitochondria–nuclear communication represents a fundamental inter-organellar process essential for maintaining cellular homeostasis and balancing survival–death signaling under cellular stress [133].

To date, studies on mitochondria–nucleus communication have primarily focused on signals sent from stressed mitochondria to the nucleus and the subsequent cellular responses, as well as on nuclear-to-mitochondrial communication in the context of DNA damage and repair. However, particularly during aging, this interaction can be seen as a dynamic, bidirectional dialogue with feedback mechanisms extending beyond these two organelles and influenced by various physiological cues [134].

In neuronal cells, the relationship between mitochondria and the nucleus is especially intricate. Neurons rely heavily on oxidative phosphorylation (OXPHOS), making them particularly susceptible to oxidative stress caused by ROS production. Given that OXPHOS components are encoded by both mitochondrial and nuclear genomes, while the “ROS defense system” is exclusively nuclear-encoded, a well-coordinated supply of these factors is essential for sustaining ATP production and maintaining neuronal integrity. Additionally, the unique and highly specialized structure of neurons necessitates a continuous supply of nuclear-encoded mitochondrial proteins to support their extensive mitochondrial network [135].

Given the central role of mitochondria in cellular metabolism, it is unsurprising that metabolites function as mitochondrial messengers capable of communicating with the nucleus, thereby rewiring key bioenergetic and stress-response signaling pathways [136]. For instance, a decline in ATP production increases the AMP/ATP ratio, activating the AMPK pathway. This pathway regulates crucial metabolic and cellular processes, including lipid and glucose metabolism, mitochondrial dynamics, autophagy, and protein synthesis, while also inducing transcriptional changes to restore energy balance [137]. Similarly, the NAD+/NADH ratio reflects mitochondrial metabolic status and modulates various enzymatic reactions, influencing deacetylation processes mediated by sirtuins across multiple cellular compartments [138]. Oxygen availability is another critical factor, as mitochondria require it for ATP generation. Under hypoxic conditions, cells activate hypoxia-inducible factors (HIFs) via prolyl hydroxylase domain proteins, enabling adaptation of mitochondrial function to oxygen scarcity [139]. Furthermore, mitochondria-derived reactive oxygen species (ROS) serve not only as toxic by-products but also as essential signaling molecules, regulating hypoxic responses, immunity, and stem cell function. They may also contribute to longevity through HIF stabilization [140,141]. Additionally, metabolites produced within mitochondria, such as acetyl-CoA and tricarboxylic acid (TCA) cycle intermediates such as α-ketoglutarate, succinate, and fumarate act as signaling molecules that link energy production with gene expression and epigenetic modifications, orchestrating a comprehensive cellular response to metabolic status [142].

As previously mentioned, mitochondria become dysfunctional during aging. This dysfunction can trigger increased ROS production, mtDNA mutations, and impaired energy production, all of which might disrupt communication with the nucleus and further contribute to the aging process.

Mitochondria-nucleus contact sites, also referred to as nucleus-associated mitochondria (NAMs), represent a recently described form of inter-organellar communication, in which mitochondria are physically tethered to the nuclear envelope. These structures facilitate the local exchange of signals, proteins, and possibly metabolites, supporting the maintenance of cellular homeostasis, particularly under stress conditions.

These contacts are not randomly distributed but are mediated by conserved protein complexes that orchestrate their formation. In mammalian cells, components such as the translocator protein (TSPO) located on the mitochondrial outer membrane, and ACBD3 and PKA on the nuclear side are essential for establishing these tethers, suggesting a regulated and structured architecture. Additionally, ancient membrane pores like the voltage-dependent anion channel (VDAC) and nuclear pore complex-associated proteins have been implicated in bridging these organelles [143,144].

Functionally, mitochondria–nucleus contact sites play a crucial role in coordinating the mitochondrial retrograde response (MRR), a signaling cascade activated upon mitochondrial dysfunction. MRR promotes the transcription of nuclear genes involved in stress adaptation, mitochondrial biogenesis, and metabolic reprogramming, ultimately helping to restore cellular homeostasis [144].

In the CNS, where mitochondrial function is vital due to high energy demands and limited regenerative capacity, the role of NAMs may be particularly significant. These contacts could be essential in coordinating stress responses, regulating gene expression, and promoting neuronal survival and plasticity. Conversely, their disruption may compromise stress adaptation and contribute to the development or progression of neurodegenerative diseases. Although direct studies on NAMs within the CNS remain limited, altered mitochondrial dynamics and defective retrograde signaling, both potentially linked to NAM dysfunction, are well-established features of conditions such as Alzheimer’s disease, Parkinson’s disease, and amyotrophic lateral sclerosis.

In summary, in the CNS, mitochondrial–nuclear communication is emerging as a key player in maintaining neuronal homeostasis by coordinating energy metabolism, redox balance, and stress responses. This bidirectional dialogue ensures the proper expression, transport, and function of mitochondrial proteins, thereby preserving neuronal integrity.

### 6.2. Mitochondrial–ER, Mitochondrial–Peroxisome, and Mitochondrial–Lysosomes Communication in Aging

Recent research has highlighted the importance of specialized contact sites between mitochondria and other organelles, including the endoplasmic reticulum (ER) [145,146], lysosomes [147], and peroxisomes [148] in maintaining cellular homeostasis and regulating metabolic processes.

Mitochondria-Associated Membranes (MAMs), the contact points between mitochondria and the ER, are essential for calcium homeostasis, lipid transfer, and mitochondrial dynamics [145,149]. With aging, MAM function becomes compromised, leading to disrupted calcium signaling, increased ER stress, and mitochondrial dysfunction [149]. These alterations contribute significantly to the pathogenesis of age-related neurodegenerative diseases, such as Alzheimer’s and Parkinson’s [149].

Mitochondria–lysosome contacts (MLCs) have also emerged as critical regulators of mitochondrial quality control and cellular metabolism [147,150]. During neuronal aging, MLC efficiency declines, resulting in impaired mitophagy and the accumulation of dysfunctional mitochondria. This accumulation exacerbates oxidative stress and inflammation—hallmarks of aging in the CNS [147].

The interaction between mitochondria and peroxisomes is also affected by the aging process. These organelles cooperate in fatty acid oxidation and reactive oxygen species (ROS) metabolism [148,151]. Age-related alterations in mitochondria–peroxisome interactions lead to metabolic imbalances and increased oxidative stress in neurons, contributing to the progression of neurodegenerative disorders [151].

Mitochondria–organelle contact sites not only facilitate the exchange of lipids, calcium, and signaling molecules but also provide structural and spatial cues that influence mitochondria-derived vesicles (MDVs) biogenesis [152]. MDVs selectively remove damaged mitochondrial components for lysosomal degradation [153]. With age, MDV formation and trafficking become less efficient, leading to the accumulation of oxidized proteins and lipids within mitochondria [154]. This impairment is particularly relevant in Parkinson’s disease, where mutations in PINK1 and PARKIN affect both mitophagy and MDV formation [153,154].

The disruption of these inter-organelle contacts during aging contributes to a cascade of cellular dysfunctions, including impaired calcium homeostasis, increased oxidative stress, accumulation of misfolded proteins, and compromised energy metabolism [145,147,148,149]. These changes collectively accelerate neuronal aging and increase susceptibility to neurodegenerative diseases.

Understanding the intricate relationships between mitochondria and other cellular organelles presents promising avenues for therapeutic interventions. Potential strategies include enhancing MAM formation, improving MLC efficiency, stimulating MDV production, and restoring proper mitochondria–peroxisome interactions [145,147,154]. Such approaches could help preserve mitochondrial function, reduce oxidative stress, and ultimately slow down the neuronal aging process.

In conclusion, mitochondrial inter-organelle contacts play a pivotal role in neuronal aging. Further research into these complex interactions will not only deepen our understanding of age-related neurodegeneration but also pave the way for novel therapeutic strategies to promote healthy brain aging.

## 7. Neurodegenerative Diseases and Mitochondrial Molecular Targets

### 7.1. Alzheimer’s Disease (AD)

Alzheimer’s disease (AD) is the most common progressive neurodegenerative disorder worldwide, mainly affecting individuals over 65 years old [155].

The neuropathological hallmarks of AD include the abnormal accumulation and aggregation of amyloid-β (Aβ) peptides in the extracellular compartment of the CNS forming so-called senile plaques (SPs), alongside the intracellular deposition of neurofibrillary tangles (NFTs), containing hyperphosphorylated tau (p-tau) protein [156]. Importantly, both Aβ plaques and p-tau NFTs are neurotoxic, synergistically triggering progressive neuronal death and brain tissue loss in the CNS. This leads to profound cortical degeneration, with clinical symptoms including severe memory loss, cognitive decline, and behavioral abnormalities in patients [157].

**Figure 2:**
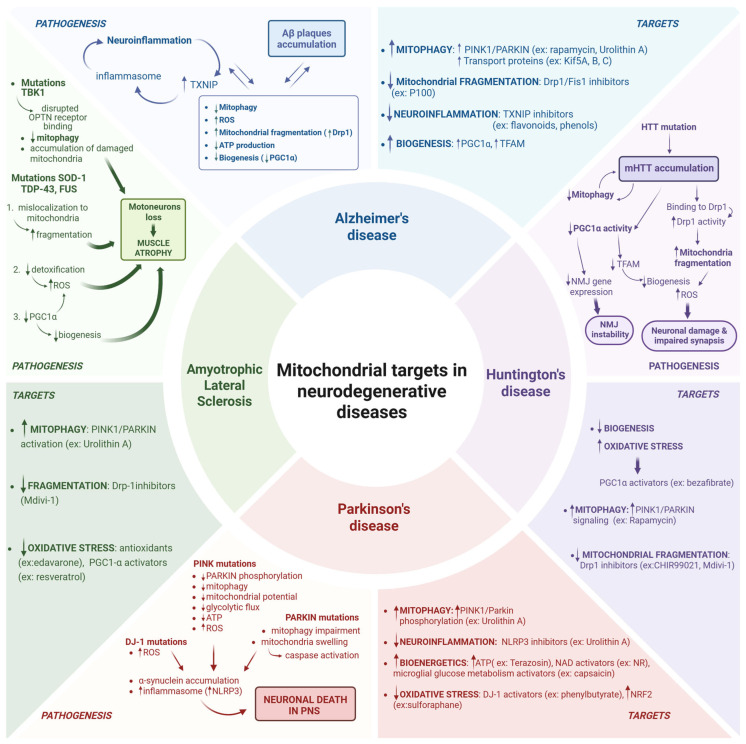
Mitochondrial targets in neurodegenerative diseases. Schematic representation of mitochondria-related pathologies and key mitochondrial therapeutic targets in Alzheimer’s, Parkinson’s, Huntington’s disease, and amyotrophic lateral sclerosis. Figure created with Biorender.

Key contributors to the neurodegenerative process in AD include neuroinflammation, mitochondrial dysfunction, excitotoxicity, and oxidative stress. Among these, mitochondrial impairment has been proposed as an early metabolic alteration and a major feature of the disease, playing a pivotal role as both a driver and predisposing factor in the onset and/or progression of AD (Figure 2) [158].

More specifically, the aberrant accumulation of neuroinflammatory signals and p-tau NFTs in close proximity to mitochondria disrupts the mitochondrial membrane potential, triggering excessive production of reactive oxygen species (ROS). The resulting oxidative stress further compromises mitochondrial function [159], and triggers oxidation and denaturation of proteins, lipids, and nucleic acids within CNS cells [160]. This cascade exacerbates the accumulation of Aβ and p-tau, which, in turn, further induce mitochondrial dysfunction and oxidative stress, thus creating a vicious cycle (Figure 2).

To date, numerous studies have demonstrated significant alterations affecting the content of various proteins involved in mitophagy, mitochondrial dynamics, and mitochondrial biogenesis in AD [157]. In this section, we summarize the principal mitochondria-targeted therapeutic strategies for the treatment of AD (Figure 2):

#### 7.1.1. Enhancing Mitophagy

As discussed previously, impaired mitophagy is responsible for the excessive accumulation of damaged mitochondria observed in the brain tissue of both transgenic mouse models of AD and human AD patients. Specifically, in AD there is a reduction in PINK1, ULK1, BNIP3L, which reduces mitophagy and triggers the accumulation of damaged mitochondria [161]. Moreover, multiple familial gene mutations such as Presenilin-1 (PSEN1) and Amyloid Precursor Protein (APP) that trigger AD are characterized by elevated lysosome pH which compromises autolysosome function and leads to the accumulation of toxic protein aggregates, such as amyloid-beta (Aβ) [162]. This, coupled with axonal transport defects, results in mitophagosomes and toxic proteins accumulating in neurites rather than reaching degradative lysosomes in the soma. Several therapeutic approaches have been proposed to restore mitophagy:

A. Activation of the PINK1/PARKIN pathway: Upregulation of PINK1 or PARKIN content has shown neuroprotective effects and therapeutic potential in the treatment of AD [163]. In microglia, it was demonstrated to significantly reduce neuroinflammation and the levels of insoluble Aβ content, ameliorating the cognitive deficits in the AD mouse model [24]. Importantly, restoration of mitophagy by oral administration of drugs such as Urolithin A and Rapamycin, which promote mitophagy, were effective in improving the cognitive deficits in both the nematode and rodent models of AD, decreasing Aβ plaques, and promoting the elimination of tau hyperphosphorylation [25,164,165].
B. Enhancing mitochondrial transport proteins: A recent study found that in AD, the presence of Aβ oligomers (Aβo) and amyloid precursor protein-C terminal fragments (APP-CTFs) alters the expression of several mitochondrial transport proteins, including SNPH and Miro1; adapter proteins such as TRANK1 and TRAK2; and components of the dynein and kinesin motor complexes, including Kif5A, Kif5B, and Kif5C. These proteins are crucial for axonal mitochondrial transport supporting mitophagy. Disruptions in their function contribute to impaired mitophagy and disease progression and restoring those genes showed improvement in AD pathogenesis [166,167].

#### 7.1.2. Decreasing Mitochondria Fragmentation

Dysregulated mitochondrial dynamics compromise neuronal function via neuroinflammatory pathways [157]. Indeed, mitochondrial fragmentation due to excessive mitochondrial fission has been shown to be present in the early stages of AD development in mice [168]. Mechanistically, Aβ and p-tau accumulation synergistically induce ROS overproduction, which activates Drp1 and its cofactor Fis1, directly driving pathological mitochondrial hyper-fission, resulting in impaired mitochondrial trafficking to synapses and significantly reduced ATP production at the synaptic level, directly impairing synaptic transmission efficacy and hence leading to neuronal functional collapse [169]. Recent therapeutic strategies have targeted this pathway to mitigate mitochondrial fragmentation:

A. Inhibition of Fis1 and Drp1: Biochemical assessments of peripheral blood from AD patients showed significantly high levels of the mitochondrial fission factor Fis1, with post-mortem brain specimens further revealing upregulated Drp1 expression in AD cases [170,171]. P110 treatment disrupts Drp1/Fis1 interaction without affecting the interaction of Drp1 with its other adaptors, which results in reduced Aβ deposition and improved behavioral deficits [172].
B. Promotion of mitochondria fusion via OPA1 and Mfn2: Han et al. recently revealed a marked downregulation of mitochondrial fusion regulators OPA1 and Mfn2 in AD groups compared to non-AD controls [173].
C. Reducing neuroinflammation: The higher expression of Drp1 and TXNIP during AD are also involved in early inflammatory responses in oligodendrocytes and microglia of AD animal models, triggering NLRP3 inflammasome activation and caspase-3 cleavage, which, in turn, amplify neuroinflammatory cascades [174,175]. These pathological processes exacerbate Aβ deposition and tau-mediated neurodegeneration, ultimately leading to neuronal death or functional deficits. Some flavonoids and phenols (e.g., rosmarinic acid, rutic, puerarin) have been proposed as potent TXNIP inhibitors [176].

#### 7.1.3. Enhancing Biogenesis

Mitochondrial biogenesis is essential for maintaining neuronal energy homeostasis and function. In Alzheimer’s disease (AD), accumulating evidence suggests that this process is markedly impaired, contributing to mitochondrial dysfunction and subsequent neurodegeneration. Evidence shows that brains from AD patients have a decreased expression of genes associated with mitochondrial biogenesis, including peroxisome-proliferator activated receptor coactivator-1 (PGC-1α), nuclear respiratory factor 1/2 (NRF1/2), and mitochondrial transcription factor A (TFAM) [177]. Accordingly, restoring or enhancing mitochondrial biogenesis has emerged as a promising therapeutic strategy in the context of AD.

A. Activation of PGC-1α: PGC-1α protein levels are inversely proportional to the concentration of Aβ in the CNS in AD making it a promising therapeutic target for AD [178]. Consistently, the overexpression of PGC-1α via adeno-associated virus in the brain of the AD murine transgenic model APP23 is sufficient to enhance the transcription of growth factors and to blunt Aβ-mediated neuroinflammation and neuronal death, leading to reduced-amyloid production and neuronal loss [179], together with decreased mitochondrial damage, thus restoring AD cognitive deficits [180].
B. Enhancing TFAM: Human mitochondrial transcription factor A (hTFAM) activation was shown to protect mtDNA, reduce oxidative damage and intracellular Aβ, and improve cognitive function in both mouse and human AD models, making it a promising therapeutic target [181].

### 7.2. Parkinson’s Disease (PD)

Parkinson’s disease (PD) is the most common neurodegenerative disorder of aging and the second most common brain aging disorder [182]. This syndrome is mainly characterized by the loss of dopaminergic neurons in the substantia nigra pars compacta (SNpc), thus leading to a dopamine deficiency in the basal ganglia. Lewy bodies are the, primary protein aggregates in PD that are mainly composed of toxic α-synuclein (α-syn) (or its phosphorylated form), ubiquitin, neurofilaments, and molecular chaperones, which are detrimental to neurons [183,184]. PD presents with a range of symptoms, including key motor impairments, such as bradykinesia, hypokinesia, resting tremor, muscle rigidity, and postural instability, as well as non-motor issues like autonomic dysfunction, sleep disturbances, depression, and cognitive decline. About 10% of cases are familial, indicating a genetic component, while the remaining 90% are sporadic, likely resulting from environmental factors or unknown genetic influences. Mitochondrial deficits and specifically reduced mitophagy are evident in both sporadic and familial PD.

In four sporadic cases of Parkinsonism, exposure to MPTP (1-methyl-4-phenyl-1,2,3,6-tetrahydropyridine) led to approximately 60% and 65% deficiency in Complex I and Complex II, respectively, in dopaminergic neurons of the substantia nigra [185]. Several mitochondrial-related genes have been identified as being associated with PD in different meta-analyses published, such as RAB7L1, MCCC1, ACMSD, and LRRK2 [186].

On the other hand, several genes have been identified as being involved in causing familial PD, that are linked with mitochondrial dysfunction. Among them, the PRKN (Parkin) gene and PINK1 genes are the most common cause of autosomal recessive PD [187]. Loss of function of PRKN contributes to mitochondrial swelling, cytochrome c release, and caspase-3 activation, which triggers cellular death [188], while mutations in PINK1 have been correlated with reduced mitochondrial membrane potential, abnormal morphology, decreased Complex I and IV activity, impaired mitochondrial transport, reduced ATP, and increased ROS production, triggering apoptosis and deficient synaptic transmission (Figure 2) [189,190].

In this section, we summarize the principal mitochondrial-targeted therapeutic strategies for treatment, depending on the specific gene mutation of PD (Figure 2):

#### 7.2.1. Boosting Mitophagy

The association of mutations in the PRKN and PINK1 genes with PD strongly suggests that impaired mitophagy and the resulting accumulation of damaged mitochondria are key factors in the disease’s pathogenesis [191]. PRKN mutations impair Parkin’s E3 ligase activity, whereas PINK1 mutations lead to a defective accumulation of PINK1 on the outer mitochondrial membrane (OMM) or to loss of its kinase function, thus preventing Parkin activation. As a result, damaged mitochondria are not cleared and accumulate, triggering ROS production and release of DAMPs (such as mtDNA), which activate inflammatory pathways. Thus, boosting mitophagy to promote the clearance of damaged mitochondria could be a good strategy for preventing disease progression:

A. Activation of PINK/Parkin signaling pathway for preventing α-syn accumulation: The pathological accumulation of phosphorylated α-syn is a key contributor to age-dependent neurodegeneration in PD [183]. In aging primates’ brains, reduced expression of Parkin and a decline in its PINK1-dependent phosphorylation contribute to substantia nigra neurodegeneration. Phosphorylated Parkin plays a critical role in neuroprotection by promoting the clearance of α-syn. Indeed, reintroducing wild-type (WT) Parkin expression, which is capable of being phosphorylated by PINK1, reduced the accumulation of pα-syn [16]. This research highlights the crucial role of Parkin phosphorylation in the development of PD, pointing to it as a potential avenue for new treatment strategies.
B. Promoting mitophagy in microglia and reducing neuroinflammation through Urolithin A (UA): Microglial-mediated neuroinflammation and mitophagy are closely interconnected. In PD murine models, neuroinflammasome pathways, particularly NLRP3, are highly activated, a response amplified by ROS generated from dysfunctional mitochondria. Urolithin A (UA), a naturally occurring compound, has been shown to promote mitophagy, restore mitochondrial function, and suppress NLRP3 inflammasome activation in PD mouse models [192]. UA administration in aged humans also effectively reduced neuroinflammation markers, ameliorated mitochondrial function, and improved muscle strength and performance, suggesting its potential as a therapeutic strategy [193,194].

#### 7.2.2. Boosting Bioenergetics

Recent evidence highlights disruptions in astrocyte and glial cell energy metabolism, marked by altered glycolysis and oxidative phosphorylation (OXPHOS) patterns, in both aging and PD models. PD-linked gene mutations may alter the metabolic profile of astrocytes, with mutant α-synuclein contributing to bioenergetic decline by disrupting lipid catabolism, impairing cellular energy homeostasis, and reducing glutamate uptake efficiency [195]. Thus, boosting bioenergetics may be a promising strategy to improve PD symptoms:

A. Rescue microglial energy metabolism: Considering the major role of microglial glucose metabolism in neuroinflammation, manipulating microglial glucose metabolism has been proposed as a potential therapeutic approach. Compounds such as capsaicin preserve dopamine neurons degeneration, blocking the activity of glial cells in inducing oxidative stress and neuroinflammation [196]. Particularly, capsaicin rescues microglial energy metabolism by increasing TRPV1 and suppressing NADPH oxidase-driven ROS production. This protects nigrostriatal dopaminergic neurons via inhibition of glial activation-mediated oxidative stress.
B. Increasing NAD+ levels: A significant decline in the NAD+/NADH ratio supports the reduction in ETC enzyme activity in mitochondria during aging and in pathologies such as PD. A Phase I trial (NCT03816020) with nicotinamide riboside (NR) showed elevated NAD+ levels in PD patients, enhancing mitochondrial and lysosomal function, and reducing inflammatory cytokines in both serum and CSF [197]. This study highlighted that 1000 mg/day for 30 days is safe, increases brain NAD+, and mildly improves clinical symptoms and cerebral metabolism in early-stage PD.
C. Increasing ATP levels: Impaired glycolytic flux in PD triggers a reduction in ATP levels. Terazosin enhanced ATP via phosphoglycerate kinase 1 (PGK1) which stimulates glycolysis and increases brain ATP production, slowing neurodegeneration in preclinical and clinical studies [198].

#### 7.2.3. Reducing Oxidative Stress

As mentioned previously, mutations in PINK-1/PRKN lead to accumulation of damaged mitochondria, which induces excessive reactive oxygen species (ROS) production. This, together with impaired antioxidant defenses, triggers oxidative stress in PD, contributing to dopaminergic neuron degeneration. To reduce oxidative stress, several strategies are being considered:

A. Increasing DJ-1 levels: Among the endogenous cytoprotective pathways, the DJ-1 protein (encoded by the *PARK7* gene) has emerged as a crucial regulator of cellular redox homeostasis. DJ-1 mutations are linked to autosomal recessive-early onset PD and have been shown to compromise mitochondrial function and antioxidant defense mechanisms. Under mitochondrial stress, DJ-1 translocates into mitochondria, where it assists protein trafficking and regulates mitochondrial metabolism. Importantly, DJ-1 also acts as a sensor and scavenger of oxidative stress, participating in the detoxification of ROS and maintaining neuronal viability [199]. Loss of DJ-1 function increases susceptibility to neurotoxins, impairs motor coordination, and shortens lifespan in animal models, emphasizing its critical role in protecting neurons from oxidative damage [200]. Phenylbutyrate is a histone deacetylase inhibitor that increases DJ-1 expression ~2–3-fold in neurons and mouse brains. In cell and mouse models of PD (including MPTP-toxicity and α-synuclein aggregation models), phenylbutyrate reduces oxidative stress, decreases α-synuclein aggregates, and preserves motor/cognitive function [201].
B. Nrf2 Pathway Activation: Nrf2 is an important regulator of antioxidant expression, such as HO-1, NQO1, SOD. A novel multitarget compound that exhibits NRF2 induction activity and MAO-B selective inhibition, combined with anti-inflammatory, antioxidant, and blood–brain barrier permeation properties, has been recently developed and has benefits in PD [202]. Moreover, sulforaphane is an interesting component derived from glucoraphanin and found in many vegetables such as broccoli and cauliflower that activates the Nrf2 pathway. Oral consumption of broccoli leads to a rapid increase in plasma sulforaphane levels within hours. Once absorbed, sulforaphane efficiently crosses the blood–brain barrier, providing neuroprotective benefits against both acute brain injury and chronic neurodegenerative diseases, such as PD [203].

### 7.3. Huntington’s Disease (HD)

HD is a progressive, autosomal dominant, hereditary neurodegenerative disorder, globally affecting 5–10 individuals per 100,000 people, and is therefore the most common hereditary neurodegenerative disease [204]. HD is caused by a mutation in the huntingtin (HTT) gene on chromosome 4 [205] encoding the HTT protein, which plays a crucial role in the CNS contributing to several cellular functions, including neurogenesis, embryonic development, vesicle trafficking, autophagy regulation, autophagosome transport in neurons, gene expression, and cell signaling [206]. Importantly, HTT is also essential for mitochondrial functions, including mitochondrial transport, localization, fusion, fission, and maintenance of the mitochondrial membrane potential [207].

In HD, mutation in the HTT gene involves the amplification of a cytosine-adenine-guanine (CAG) trinucleotide repeat encoding the amino acid glutamine (Gln), in the first exon of the gene, with 36 or more repetitions. This expansion leads to the production of a mutant protein (mHTT9) with a long poly-glutamine (polyQ) stretch, resulting in protein misfolding and progressive accumulation and aggregation in the cytoplasm and nucleus of cells [208]. In particular, in the brain, the accumulation of mHTT interferes with mitochondrial functions, leading to bioenergetic deficits and an increase in oxidative stress, causing neuronal damage, impaired calcium homeostasis, and altered mitochondrial dynamics [209]. Furthermore, mHTT disrupts synaptic morphology and function, as well as neuronal proteostasis, ultimately contributing to neuronal loss and nerve damage [210].

Given that HD affects different regions of the brain, its typical clinical manifestations include motor and cognitive disorders, as well as psychiatric disturbances. Additionally, motor abnormalities resulting from striatal dysfunction, which is characterized by progressive, involuntary dance-like movements, are also observed [211].

In recent years, an increasing body of evidence has highlighted the critical role of mitochondrial dysfunction in the etiology of HD, making it a significant study area for possible therapeutic approaches. However, the precise pathogenic pathways involved have not yet been thoroughly elucidated, even though the neurotoxic effects of mHTT are mainly mediated by the induction of aberrant mitochondrial energy metabolism and HD-related neuronal dysfunction.

In this section, we describe the principal mitochondrial-targeted therapeutic strategies for the treatment of Huntington’s disease (Figure 2):

A. Enhancing mitochondrial biogenesis by increasing PGC-1α: Research evidence indicates that mutant huntingtin (mHTT) directly interacts with the promoter region of PGC-1α and interferes with the CREB/TAF-dependent transcriptional pathway, leading to reduced transcriptional activity. This downregulation impairs the expression of key downstream targets, including mitochondrial transcription factor A (TFAM)**,** thereby decreasing biogenesis and contributing to mitochondrial dysfunction, increased oxidative stress susceptibility, and progressive neuronal degeneration [212]. Additionally, mHTT-mediated suppression of PGC-1α affects genes critical for the maintenance of neuromuscular junction stability [213]. Oral administration of bezafibrate restored levels of PGC-1α and downstream targets such as NRF-1 and TFAM, showing improvements in an HD mouse model by increasing mitochondrial biogenesis and reducing lipid accumulation [209,214].
B. Enhancing mitophagy through PINK activation: Research studies have demonstrated that mHTT is also involved in the process of mitophagy. mHTT inhibits the commitment of mitophagy receptors, such as p62 and OPTN, to damaged mitochondria and impairs their interaction with LC3, thereby blocking autophagosome formation and disrupting mitophagy initiation [207]. mHTT also impairs Parkin recruitment to mitochondria and inhibits PINK1 accumulation on the outer mitochondrial membrane [207,215]. This results in defects in mitophagy, where a large number of dysfunctional mitochondria are not removed in time and excessively accumulate in neuronal cells, inducing a vicious cycle [216]. It was observed that the overexpression of PINK1 in a *Drosophila* model of HD resulted in the amelioration of Parkin-mediated mitophagy defects and the attenuation of mHTT-induced neurotoxicity, promoting neuronal activity and protecting neuronal integrity [215]. Consequently, mitophagy serves to protect neurons from damage in HD, and a deficiency in this process contributes to further malignant deterioration of HD pathology [157].
C. Decreasing mitochondria fragmentation through Drp1 inhibition: The accumulation of mHTT in neuronal cells in the brains of HD patients has been shown to induce mitochondrial hyper-fragmentation and to affect the mitochondrial respiratory chain complex by disrupting the balance of mitochondrial dynamics, which, in turn, induces impairment of mitochondrial function [217]. Moreover, in the brains of HD patients, the expression levels of the mitochondrial fission/fusion proteins Drp1, Fis1, and Mfn are significantly altered [218]. Sawant et al. demonstrated that mHTT is able to bind to the proteins Mfn and Drp1. This binding enhances the activity of Drp1 and, together with a downregulation of fusion proteins such as Mfn1/2 and OPA1, results in mitochondrial fusion and fission imbalances. Fragmented mitochondria are less efficient at ATP production and produce more ROS. Moreover, this fragmentation triggers mitochondrial distribution abnormalities and mitochondrial axonal transport defects, which ultimately lead to impaired synaptic plasticity [219]. Coherently, inhibition of Drp1 has been demonstrated to restore mitochondrial and neuronal dysfunction in mHTT-induced HD animal models. In particular, treatment with CHIR99021 stabilizes calpastatin (CAST), thereby inhibiting calpain activation and preventing Drp1-mediated mitochondrial fragmentation, reducing HD-associated neuropathology and behavioral defects [157,220].

### 7.4. Amyotrophic Lateral Sclerosis (ALS)

Amyotrophic lateral sclerosis (ALS) represents the most prevalent subtype of motor neuron diseases (MNDs), a group of neurodegenerative disorders characterized by the involvement of both upper and lower motor neurons. ALS is a relentlessly progressive condition marked by the degeneration and eventual loss of motor neurons, leading to increasing muscle weakness and, ultimately, respiratory compromise [221].

Among the key pathological features of ALS, mitochondrial dysfunction plays a central role in disease onset and progression. Mitochondrial impairments in ALS trigger altered dynamics, defective mitophagy, impaired bioenergetics, increased oxidative stress, and defective biogenesis (Figure 2). Several ALS-linked genes are directly involved in these mitochondrial processes [222,223]. The most frequently implicated include the hexanucleotide repeat expansion in the chromosome 9 open reading frame 72 (C9ORF72), Tank-binding kinase 1 (TBK1), superoxide dismutase 1 (SOD1), TAR DNA-binding protein 43 (TARDBP, encoding TDP-43), fused in sarcoma (FUS), and CHCHD10. Different strategies are known to ameliorate disease progression targeting mitochondria-related genes (Figure 2):

A. Promoting mitophagy: In ALS, ninety distinct mutations in TBK1 are linked to ALS and frontotemporal dementia, including missense mutations that disrupt TBK1’s ability to dimerize and associate with the mitophagy receptor optineurin (OPTN), thereby impairing autophagy/mitophagy initiation and mitochondrial clearance. Additionally, mutations in C9ORF72 disrupt endosomal and autophagic trafficking, leading to defective clearance of damaged mitochondria and accumulation of dysfunctional mitochondria [224]. Moreover, TDP-43 and FUS aggregates sequester essential mitochondrial proteins and impair mitochondrial transport along axons, further compounding mitophagy defects [225,226]. While urolithin A has shown promise as treatment for improving motor dysfunction by activating mitophagy [227], rapamycin, which also increases mitophagy by inhibiting mTOR, only showed positive effects in decreasing neuroinflammation in clinical studies, even though preclinical studies have shown reduced TDP-43 aggregation and improved motor neuron function in ALS [228]. The same has been observed with trehalose, another autophagy activator, for which preclinical studies showed promising results, but in large-scale trials did not demonstrate efficacy in patients [229]. However, trehalose ameliorates the pathogenesis of some subgroups, suggesting that more personalized research is needed.
B. Reducing fragmentation though downregulation of Drp-1: Mutant SOD1, TDP-43, and FUS mislocalize to mitochondria and promote excessive fission via Drp1 upregulation and OPA1/Mfn2 downregulation [230,231]. On the other hand, CHCHD10 mutations affect cristae structure and fusion [232]. Excessive Drp1-mediated mitochondrial fission contributes to mitochondrial fragmentation, which impairs ATP synthesis and facilitates cytochrome c release, triggering apoptotic pathways and motor neuron death. Suppression of the Drp-1 cascade prevents ALS-related symptoms [233].
C. Reducing oxidative stress and ROS production: Mitochondria in ALS show increased ROS production, partly due to SOD1 mutations, which impair detoxification of superoxide radicals, and TDP-43, which inhibits mitochondrial antioxidant responses. Increased mitochondrial ROS also trigger activation of the mitochondrial permeability transition pore (mPTP), promoting mitochondrial swelling and release of pro-apoptotic factors [234,235]. Targeting ROS with antioxidants such as edaravone, which is FDA-approved for ALS [236], helps scavenge ROS, thereby reducing oxidative stress and protecting mtDNA and mitochondrial function.
D. PGC-1α activation: ALS models show reduced PGC-1α levels and downstream regulators such as NRF1 and TFAM, limiting mitochondrial renewal and repair [213], which further exacerbates energy failure and oxidative stress. PGC-1α also regulates the expression of key antioxidant enzymes and mitochondrial fusion/fission proteins; thus, its reduction amplifies mitochondrial vulnerability in ALS [237]. Activation of PGC-1α by resveratrol ameliorates mitochondrial function in ALS animal models [238].

Nevertheless, the majority of sporadic ALS cases do not involve mutations in these known ALS-associated genes. Emerging evidence suggests that genetic predispositions, environmental exposures, for example, heavy metals or organic chemicals such as pesticides and solvents, traumatic brain injury, as well as lifestyle and age-related factors, may all contribute to the etiology of ALS [223] but more research is needed to unravel the molecular pathogenesis underlying these factors.

## 8. Challenges and Future Directions

Emerging therapeutic strategies aimed at restoring mitochondrial health offer promising avenues for intervention. Lifestyle factors, including diet and exercise, also influence mitochondrial aging. Many of the neurodegenerative and age-related diseases described in this review exhibit abnormalities in mitochondrial biogenesis, mitophagy, OXPHOS reduction, and mitochondrial dynamics, which ultimately influence neuronal function and lead to cognitive deficits.

Other central nervous system (CNS) pathologies involve mitochondrial disorders, yet these conditions are not primarily associated with the aging process. This is the case for multiple sclerosis (MS), spinocerebellar ataxia (SCA), epilepsy, traumatic brain injury (TBI), stroke, and HIV-associated neurocognitive disorder (HAND). For example, MS in an autoimmune disease that typically affects young adults between the ages of 20 and 40, in which the immune system mistakenly attacks the myelin sheath covering neurons, leading to inflammation and progressive neuronal degeneration [239]. Unlike classical neurodegenerative disorders such as Alzheimer’s disease, Parkinson’s disease, Huntington’s disease, and ALS, which are prominently defined by the accumulation of misfolded and aggregated proteins (such as amyloid-β, tau, α-synuclein, TDP-43, or mutant huntingtin), MS is not driven by pathological protein aggregation. Instead, mitochondrial impairment in MS is closely linked to chronic inflammation, immune cell activation, and oxidative stress [240]. As such, the mechanisms of neurodegeneration in MS differ substantially from those observed in protein aggregation-associated disorders, despite overlapping mitochondrial disturbances.

For neurodegenerative diseases such as Alzheimer’s disease, Parkinson’s disease, Huntington’s disease, and ALS, an increasing number of studies exploring novel therapeutic and lifestyle-modifying interventions have demonstrated the potential benefits of mitigating mitochondria-dependent oxidative stress, enhancing mitochondrial biogenesis, CNS metabolism and restoring mitophagy through strategies like fasting and physical exercise as well as pharmacologically, with the so-called mitophagy enhancers, such as urolithin A and rapamycin [24,241], and antioxidants such as edaravone, which is FDA-approved for ALS [236].

Unfortunately, many drugs that improved mitochondrial function in preclinical trials have failed to show efficacy or have raised safety concerns in clinics. One example is Mdivi-1, a Drp-1 inhibitor that showed protective effects by mitigating mitochondrial dysfunction and synaptic impairment in HD [242] and by improving mitochondrial morphology and survival in ALS mouse models [243]. However, recent studies have shown that Mdivi-1 also interferes with mitochondrial bioenergetics and complex I function, which is associated with increased ROS production and reduced ATP generation efficiency [244].

Other treatments targeting oxidative stress, such as Coenzyme Q10 or N-acetylcysteine (NAC) demonstrated promising effects in preclinical studies of HD and ALS, where they improved survival and slowed functional decline. However, clinical trials have not provided consistent evidence of their efficacy [245,246]. Another example is rapamycin, an mTOR inhibitor that activates autophagy and showed promising results in preclinical studies of AD by reducing amyloid-beta plaques and tau protein tangles [164]. However, in a recent Phase I pilot clinical trial, rapamycin was undetectable in the cerebrospinal fluid of patients before and after treatment using 1 mg/day for eight weeks. Moreover, inflammatory biomarkers were even more elevated after treatment, indicating the need for further investigation into the biological effects and clinical implications of rapamycin administration [247].

The convergence of multiple pathogenic pathways, ranging from mitochondrial dysfunction and oxidative stress to impaired axonal transport, suggests a complex and multifactorial disease process in neurodegeneration. This complexity likely underlies the limited success of therapeutic strategies that target single molecular pathways. Accordingly, a combination therapy addressing multiple aspects of the disease pathology may be necessary to effectively slow or halt neurodegeneration in disease progression.

Future directions also involve utilizing advanced genetic techniques, such as antisense oligonucleotides (ASOs) for the treatment of many neurodegenerative diseases such as HD, ALS, and AD [248]. Furthermore, gene editing approaches such as CRISPR/Cas9 [249], may help restore mitochondrial health indirectly by reactivating the expression of protective genes such as PGC-1α and TFAM.

This highlights the importance of deeply dissecting the mechanisms of mitochondrial dysfunction in neurodegenerative pathogenesis to further identify novel molecular targets and guide the development of effective new therapeutics.

## 9. Conclusions

Mitochondrial dysfunction plays a pivotal role in the pathogenesis of a wide range of neurodegenerative CNS disorders. Disruptions in mitochondrial bioenergetics, dynamics, biogenesis, and quality control contribute to neuronal vulnerability, cognitive decline, and disease progression. While considerable progress has been made in elucidating the molecular underpinnings of mitochondrial dysfunction, effective translation into clinical therapies remains limited.

In this review we described how mitochondrial impairments are influenced by aging and contribute to the pathogenesis of four major neurodegenerative diseases, such as ALS, Alzheimer’s, Parkinson’s, and Huntington’s disease, in which protein aggregation is a hallmark and central pathogenic feature. We also discussed emerging therapeutic strategies targeting mitochondrial pathways, including mitophagy activation, reduction in mitochondrial fragmentation, oxidative stress mitigation, and enhancement of mitochondrial biogenesis. We highlighted that other non-aggregation CNS disorders, such as multiple sclerosis (MS), traumatic brain injury, and HIV-associated neurocognitive disorder, may require a tailored, disease-specific approach, as the mitochondrial dysfunction observed in these conditions is not the primary driver of the disease pathology.

Continued research into the interplay between mitochondrial health, neuroinflammation, and systemic metabolic status will be crucial for advancing targeted therapies. The integration of gene editing technologies, lifestyle-based interventions, and combination treatments offers promising avenues. A deeper mechanistic understanding will not only inform novel treatment strategies but also improve disease prevention and patient outcomes across a spectrum of neurological conditions.

## Data Availability

Not applicable.
